# *In planta* deglycosylation improves the SARS-CoV-2 neutralization activity of recombinant ACE2-Fc

**DOI:** 10.3389/fbioe.2023.1180044

**Published:** 2023-05-03

**Authors:** Shiva Izadi, Ulrike Vavra, Stanislav Melnik, Clemens Grünwald-Gruber, Esther Föderl-Höbenreich, Markus Sack, Kurt Zatloukal, Josef Glössl, Eva Stöger, Lukas Mach, Alexandra Castilho, Richard Strasser

**Affiliations:** ^1^ Institute of Plant Biotechnology and Cell Biology, Department of Applied Genetics and Cell Biology, University of Natural Resources and Life Sciences Vienna, Vienna, Austria; ^2^ Core Facility Mass Spectrometry, University of Natural Resources and Life Sciences Vienna, Vienna, Austria; ^3^ Diagnostic and Research Institute of Pathology, Medical University of Graz, Graz, Austria; ^4^ Pro-SPR GmbH, Alsdorf, Germany

**Keywords:** COVID-19, glycosylation, posttranslational modification, recombinant protein expression, glycoengineering

## Abstract

SARS-CoV-2 infects human cells via binding of the viral spike glycoprotein to its main cellular receptor, angiotensin-converting enzyme 2 (ACE2). The spike protein-ACE2 receptor interaction is therefore a major target for the development of therapeutic or prophylactic drugs to combat coronavirus infections. Various engineered soluble ACE2 variants (decoys) have been designed and shown to exhibit virus neutralization capacity in cell-based assays and *in vivo* models. Human ACE2 is heavily glycosylated and some of its glycans impair binding to the SARS-CoV-2 spike protein. Therefore, glycan-engineered recombinant soluble ACE2 variants might display enhanced virus-neutralization potencies. Here, we transiently co-expressed the extracellular domain of ACE2 fused to human Fc (ACE2-Fc) with a bacterial endoglycosidase in *Nicotiana benthamiana* to produce ACE2-Fc decorated with N-glycans consisting of single GlcNAc residues. The endoglycosidase was targeted to the Golgi apparatus with the intention to avoid any interference of glycan removal with concomitant ACE2-Fc protein folding and quality control in the endoplasmic reticulum. The *in vivo* deglycosylated ACE2-Fc carrying single GlcNAc residues displayed increased affinity to the receptor-binding domain (RBD) of SARS-CoV-2 as well as improved virus neutralization activity and thus is a promising drug candidate to block coronavirus infection.

## 1 Introduction

The emergence of pathogenic viruses like severe acute respiratory syndrome coronavirus 2 (SARS-CoV-2) highlights the urgent need for the development of novel preventive and therapeutic antiviral therapies to foster pandemic preparedness. SARS-CoV-2 has evolved continuously which resulted in the emergence of several virus variants that show increased infectivity. However, all known SARS-CoV-2 variants utilize angiotensin-converting enzyme 2 (ACE2) as host cell receptor (Gawish et al., 2022; Zhu et al., 2022) which makes the ACE2-spike protein interaction a central target for the development of antiviral interventions. Human ACE2 receptor decoys are therefore promising recombinant therapeutics to treat SARS-CoV-2 infections by preventing the interaction of the viral spike protein with ACE2 molecules residing on the surface of target cells. In contrast to neutralizing antibodies which are affected by escape mutations, ACE2 receptor decoys are active against all known SARS-CoV-2 variants of concern (Arimori et al., 2022; Lin et al., 2022; Monteil et al., 2022; Tada et al., 2023). In comparison to the original SARS-CoV-2 isolate, evolved variants like Omicron or Delta display enhanced and prolonged binding to ACE2 (Zhou et al., 2022), and are therefore even more susceptible to ACE2-based therapeutics (Monteil et al., 2022). Of note, administration of human recombinant soluble ACE2 (hrsACE2) was well tolerated by healthy humans in a clinical trial (Haschke et al., 2013) and 0.4 mg/kg hrsACE2 administered twice daily was successfully utilized for the compassionate-use treatment of a SARS-CoV-2 infected patient with life-threatening disease symptoms (Zoufaly et al., 2020). Monomeric soluble ACE2 has a short *in vivo* half-life, but fusion to the Fc domain of human IgG1 has been shown to increase its half-life in the circulation to approximately 30 h due to binding to the neonatal Fc receptor (FcRn) (Liu et al., 2018). Moreover, the use of Fc fusion proteins enables the tuning of effector functions either via glycoengineering of the conserved N-glycan in the Fc domain or by the introduction of point mutations to modulate Fc receptor engagement (Liu et al., 2020). Different ACE2-Fc decoys have been designed and shown to prevent infections by the parental SARS-CoV-2 strain and a broad range of variants like Omicron in animal and human tissue models, and different recombinant ACE2 variants are currently in clinical trials (Lei et al., 2020; Higuchi et al., 2021; Huang et al., 2021; Ikemura et al., 2022; Zhang et al., 2023).

In a previous study, we have shown that plant-produced recombinant soluble ACE2-Fc containing ACE2 amino acids 18-615 is functional regarding enzymatic activity, affinity to the SARS-CoV-2 receptor-binding domain (RBD) and virus neutralization (Wuhan-Hu-1 isolate) (Castilho et al., 2021). Moreover, *in vitro* enzymatic removal of N-glycans from mammalian cell-produced soluble ACE2 resulted in a SARS-CoV-2 decoy with enhanced neutralizing activity (Capraz et al., 2021). To examine whether a functionally superior ACE2-Fc decoy carrying only N-glycans consisting of a single GlcNAc residue attached to asparagine instead of canonical plant or human-like N-glycans can be produced *in planta* we carried out a transient glycoengineering study in *N. benthamiana*. Such an *in planta* produced ACE2-Fc decoy may be useful to prevent and treat coronavirus infections including those caused by emerging variants.

## 2 Materials and methods

### 2.1 Cloning

A codon-optimized (for *N. benthamiana*) DNA fragment coding for the barley α-amylase signal peptide and amino acids 18-740 of human ACE2 including the collectrin domain was synthesized (GeneArt Gene Synthesis, Thermo Fisher Scientific) and cloned into the *Age*I and *Xho*I sites of plant expression vector pEAQ-*HT* (Sainsbury et al., 2009) or the *Bsa*I sites of the magnICON vector pICH*α*26211 (Castilho et al., 2021). For expression of ST-EndoH-GFP, a codon-optimized (for *N. benthamiana*) synthetic fragment (GeneArt Gene Synthesis, Thermo Fisher Scientific) coding for amino acids 43-313 of endo-β-N-acetylglucosaminidase H (EndoH) from *Streptomyces plicatus* (P04067) was cloned into the *Bam*HI site of plant expression vector p20 (derivative of pBI101, with a cauliflower mosaic virus 35S promoter and the sequence for generation of GFP fusion proteins) (Schoberer et al., 2009) to generate p20-EndoH. The coding sequence for the N-terminal region (amino acids 1–52) of rat α2,6-sialyltransferase (Strasser et al., 2009) was cloned into the *Xba*I/*Bam*HI sites of p20-EndoH to generate p20-ST-EndoH.

### 2.2 Confocal microscopy

Leaves of 5-week-old wild-type *N. benthamiana* were infiltrated with agrobacterium suspensions (OD_600_ = 0.1) carrying the p20-ST-EndoH plasmid. Confocal images were acquired 48 h after infiltration using a Leica SP5 confocal microscope (Leica Microsystems). Samples were excited using a 488 nm laser line and collected from 500 to 530 nm as described (Schoberer et al., 2019).

### 2.3 ACE2-Fc expression and purification

ACE2-Fc was produced by agrobacterium-mediated transient expression in leaves of *N. benthamiana* ΔXT/FT (Strasser et al., 2008) or wild-type plants. Three days after infiltration (ACE2-Fc co-expressed with ST-EndoH) or 4 days after infiltration (ACE2-Fc), a total soluble protein extract was prepared and clarified by centrifugation and filtration steps as described previously (Göritzer et al., 2019). For experiments with the α-mannosidase inhibitor, 50 µM kifunensine (Santa Cruz Biotechnology) was infiltrated into leaves together with *Agrobacterium tumefaciens* carrying plasmids for transient expression of ACE2-Fc and ST-EndoH. ACE2-Fc was purified by affinity chromatography using a 5 mL HiTrap Protein A column (Cytiva) and 0.1 M glycine-HCl (pH 3.5) for elution. Eluate fractions were immediately neutralized using 2 M Tris (pH 12.0), pooled, and dialyzed against PBS (pH 7.4) at 4°C overnight using SnakeSkin Dialysis Tubing (Thermo Fisher Scientific). To isolate the dimeric fraction, affinity-purified ACE2-Fc was subjected to size exclusion chromatography (SEC) using a HiLoad 16/600 Superdex 200 pg column (Cytiva) equilibrated with PBS (pH 7.4). The purified protein was concentrated (Amicon Ultra-0.5 Centrifugal Filter Units, Millipore) and stored at −80°C until further use.

### 2.4 SDS-PAGE and immunoblotting

ACE2-Fc separated by SDS-PAGE under reducing conditions was detected by Coomassie Brilliant Blue staining or immunoblotting with anti-human IgG (H + L)-horseradish peroxidase antibody (Promega). For *in vitro* deglycosylation, purified ACE2-Fc proteins were incubated with Endoglycosidase H (EndoH) or Peptide-N-glycosidase F (PNGase F) (New England Biolabs) according to the manufacturer’s instructions and subjected to SDS-PAGE. ST-EndoH-GFP was detected on immunoblots using an anti-GFP-HRP antibody (Miltenyi Biotec).

### 2.5 SPR binding assays

Binding experiments of ACE2-Fc variants to RBD were performed using a Biacore T200 instrument (Cytiva). SEC-purified monomeric RBD (Arg319 - Lys541, Wuhan-Hu-1 sequence, with a C-terminal hexa-histidine tag) produced in HEK293 cells was available from a previous study (Klausberger et al., 2022). All measurements were conducted with a CM5 chip (Cytiva) containing 7500 resonance units of protein A immobilized via amine coupling. Prior to SPR measurements the protein concentration of each sample was verified by averaging five independent OD readings obtained with a NanoDrop spectrophotometer operated in the UV-mode (at 280 nm using sample-specific extinction coefficients). ACE2-Fc (2.63 nM) in HBS-EP buffer (0.01 M HEPES pH 7.4, 0.15 M NaCl, 3 mM EDTA and 0.005% (v/v) Surfactant P20, 0.2 µm filtered and degassed) was used as capture solution. 200 nM RBD and four 2.5-fold serial dilutions thereof (in HBS-EP) were used in single-cycle kinetic experiments. Binding affinities (K_D_) were calculated with Biacore T200 evaluation software using a 1:1 binding model. Two independent experiments were performed for two different batches of each ACE2-Fc variant (n = 4).

### 2.6 Virus neutralization assays

SARS-CoV-2 neutralization assays were performed similarly as described previously (Capraz et al., 2021) using human Calu-3 cells and the SARS-CoV-2 Delta variant. Calu-3 cells (ATCC United States) were obtained from the Core Facility Alternative Biomodels and Preclinical Imaging (Medical University of Graz, Graz, Austria). Calu-3 cells were grown in Minimum Essential Medium (MEM, Gibco 11,090-081) containing 1% penicillin/streptomycin stock solution, 1 mM L-glutamine (all from Thermo Fisher Scientific) and 10% fetal bovine serum (FBS), at 37°C and 5% CO_2_. SARS-CoV-2 Delta variant isolate (GK/478K.V1 - B.1.617. 2+ AY.x, GISAID name: hCoV-19/Austria/Graz-MUG21/2021) propagated in Vero E6 cells was preincubated with ACE2-Fc for 30 min at 37°C and subsequently used to infect Calu-3 cells with a multiplicity of infection (MOI) of 0.002. Untreated infected cells were used as controls in the assay. After incubation for 24 h at 37°C in MEM supplemented with 2% FBS, viral RNA was extracted from the culture supernatant of infected cells using the QiaAmp Viral RNA Minikit (Qiagen, Germany), according to the manufacturer’s protocol and quantified by RT-qPCR as described in detail previously (Capraz et al., 2021). One-way ANOVA was performed using GraphPad PRISM 9.5.0.

### 2.7 Glycopeptide analysis

SEC-purified ACE2-Fc was sequentially digested with chymotrypsin and trypsin and the glycopeptides were subjected to LC-ESI-MS analysis as described previously (Castilho et al., 2021). The possible glycopeptides were identified as sets of peaks consisting of the peptide moiety and the attached N-glycan varying in the number of HexNAc, hexose, deoxyhexose, and pentose residues. Manual glycopeptide searches were performed using FreeStyle 1.8 (Thermo Scientific), deconvolution was done using the extract function.

## 3 Results

**FIGURE 1:**
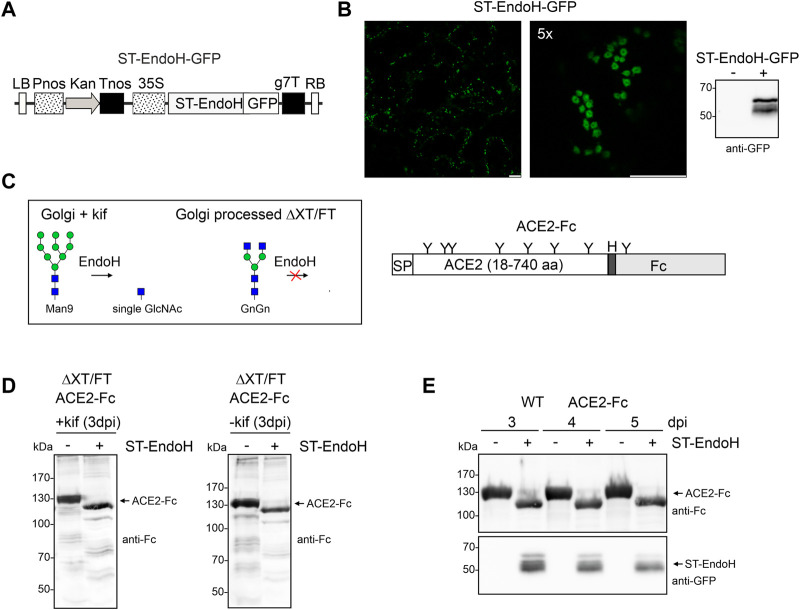
ST-EndoH-GFP is targeted to the Golgi and cleaves off N-glycans from co-expressed ACE2-Fc. **(A)** Schematic representation of the ST-EndoH-GFP expression construct. LB: left border; Pnos: nopaline synthase gene promoter; Kan: neomycin phosphotransferase II gene; Tnos: nopaline synthase gene terminator; CaMV35S: cauliflower mosaic virus 35S promoter; ST-EndoH: *Streptomyces plicatus* endo-β-N-acetylglucosaminidase H catalytic domain; GFP: green fluorescent protein; g7T: agrobacterium gene 7 terminator; RB: right border. **(B)** Confocal images of ST-EndoH-GFP expressed in leaf epidermal cells of *N. benthamiana* wild-type plants. Scale bars = 10 µm. The left image shows a larger area of the leaf with several individual cells, the right image (zooming factor 5) shows a single cell with characteristic doughnut-like Golgi bodies. The immunoblot was probed with anti-GFP antibodies. **(C)** Illustration of EndoH activity *in planta* (green symbol mannose; blue symbol GlcNAc, for structural details see http://www.functionalglycomics.org/) and of the expressed ACE2-Fc protein. SP: signal peptide, “H” hinge region, “Y” position of N-glycans. **(D)** Immunoblot detection of ACE2-Fc expressed in the ΔXT/FT line in the absence (-kif) or presence (+kif) of kifunensine and ST-EndoH-GFP (−/+ ST-EndoH). **(E)** Immunoblot detection of ACE2-Fc and ST-EndoH-GFP expressed in wild-type (WT) *N. benthamiana* plants; samples were harvested at the indicated days post infiltration (dpi).

To produce glycoengineered ACE2-Fc with N-glycans consisting of single GlcNAc residues, the catalytic domain of endo-β-N-acetylglucosaminidase H (EndoH) from *S. plicatus* was fused to the *trans*-Golgi targeting and retention sequence of rat α2,6-sialyltransferase (ST) (Strasser et al., 2009). In addition, GFP was attached to the C-terminus of ST-EndoH to enable detection on immunoblots and analysis of its subcellular localization in plant cells (Figure 1A). Confocal microscopy showed that transiently expressed ST-EndoH-GFP was detected exclusively in puncta resembling Golgi bodies in *N. benthamiana* leaf epidermal cells (Figure 1B). We co-infiltrated the ACE2-Fc expression cassette encoding amino acids 18-740 of the extracellular ACE2 domain (Figure 1C) together with ST-EndoH-GFP and the α-mannosidase inhibitor kifunensine to achieve the accumulation of mannosidic N-glycans that can be cleaved by EndoH in the Golgi apparatus. Immunoblot analysis of total protein extracts obtained 3 days after infiltration showed that ACE2-Fc migrated faster after synthesis in the presence of ST-EndoH-GFP which is indicative of *in vivo* N-glycan removal (Figure 1D). When ST-EndoH-GFP was co-infiltrated without kifunensine, a similar shift in mobility was detectable indicating that the majority of the ACE2 N-glycans are removed, probably in the *cis*/medial-Golgi, before their processing into EndoH-resistant complex N-glycan structures can occur (Figure 1C). These initial experiments were carried out with ΔXT/FT plants that are glycoengineered to produce mainly human-like GlcNAc_2_Man_3_GlcNAc_2_ (GnGn) N-glycan structures lacking plant-specific β1,2-xylose and core α1,3-fucose residues (Strasser et al., 2008). To show that the approach is more broadly applicable, we transiently co-expressed ACE2-Fc and ST-EndoH-GFP in wild-type *N. benthamiana* without kifunensine. Also, in wild-type plants that normally produce EndoH-resistant complex N-glycans carrying β1,2-xylose and core α1,3-fucose residues, we observed a shift in mobility upon SDS-PAGE and immunoblotting of ACE2-Fc. The shift in mobility was detectable 3, 4 and 5 days after infiltration revealing that ST-EndoH-GFP remains active throughout this incubation period which is frequently used for transient protein expression in *N. benthamiana* (Figure 1E).

**FIGURE 2:**
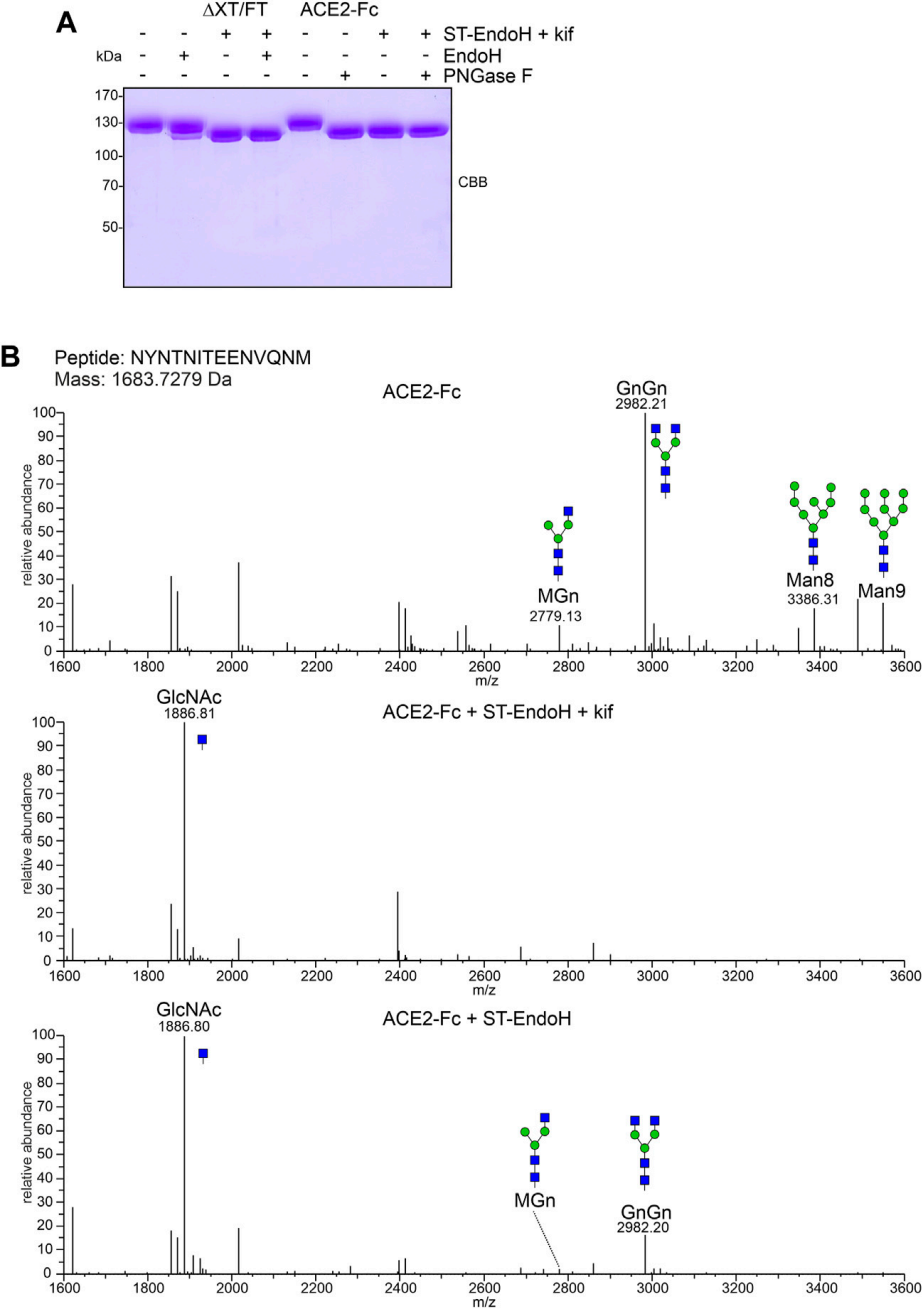
ACE2-Fc N-glycan analysis. **(A)** SDS-PAGE and Coomassie Brilliant Blue (CBB) staining of purified ACE2-Fc variants digested with EndoH or PNGase F *in vitro*. **(B)** MS spectra of the glycopeptide carrying the N53 N-glycan. The assigned N-glycan structures were labelled according to the ProGlycAn nomenclature (http://www.proglycan.com/). A cartoon illustration (green symbol mannose; blue symbol GlcNAc, for details see http://www.functionalglycomics.org/) highlights the main glycan structures detected for each peptide.

For further characterization, we purified ACE2-Fc co-expressed with ST-EndoH-GFP and kifunensine from ΔXT/FT plants by affinity and size exclusion chromatography. The overall yield after protein A purification was 80–100 μg/g leaf material with approximately 50 μg/g recovered as dimer after SEC purification which is consistent with previously reported expression levels for ACE2-Fc (Castilho et al., 2021). As a control, we produced ACE2-Fc without co-expression of ST-EndoH-GFP and subsequently digested the purified ACE2-Fc dimer *in vitro* with EndoH or PNGase F. Upon SDS-PAGE, the migration position of the *in vitro* digested ACE2-Fc proteins was comparable to the *in vivo* digested ACE2-Fc implying that the majority of ACE2 N-glycans can be removed *in planta* by ST-EndoH-GFP co-expression (Figure 2A). To confirm this, we subjected purified ACE2-Fc to proteolytic digestion and analyzed the resulting glycopeptides by mass spectrometry (MS) (Castilho et al., 2021). ACE2-Fc produced in the glycoengineered *N. benthamiana* ΔXT/FT plants carried predominantly GnGn N-glycans on six of the seven ACE2 N-glycosylation sites (Table 1). On site N690, GlcNAc_1_Man_3_GlcNAc_2_ (MGn) appeared as the most abundant N-glycan. Except for site N432 which was only glycosylated to approximately 50%, all other sites were efficiently glycosylated. By contrast, single GlcNAc residues were detected almost exclusively on all seven ACE2 N-glycosylation sites when ACE2-Fc was co-expressed with ST-EndoH-GFP in the presence of the α-mannosidase inhibitor kifunensine (Figure 2B; Table 1). In the absence of kifunensine, ACE2-Fc was also almost quantitatively deglycosylated *in vivo* and hence single GlcNAc residues were dominant on all seven N-glycosylation sites. However, small amounts of GnGn as well as other N-glycan forms were still detectable. This indicates that addition of kifunensine is required to produce homogeneously deglycosylated ACE2-Fc *in planta* (Table 1).

**TABLE 1:** ACE2-Fc N-glycans (relative amounts in %).

N-glycosylation sites							
Control	N53	N90	N103	N322	N432	N546	N690
non-glycosylated	0	0	0	2	49	3	6
single GlcNAc	1	1	0	0	0	0	2
GnGn	64	54	48	50	24	69	12
Man5-Man9	29	31	26	38	12	13	32
other N-glycans	6	14	26	10	15	15	48
total	100	100	100	100	100	100	100
ST-EndoH + kif	N53	N90	N103	N322	N432	N546	N690
non-glycosylated	1	0	0	0	54	0	0
single GlcNAc	96	100	100	100	46	100	100
GnGn	0	0	0	0	0	0	0
Man5-Man9	3	0	0	0	0	0	0
other N-glycans	0	0	0	0	0	0	0
total	100	100	100	100	100	100	100
ST-EndoH	N53	N90	N103	N322	N432	N546	N690
non-glycosylated	1	16	0	0	46	2	11
single GlcNAc	83	67	84	70	52	89	66
GnGn	13	10	12	22	1	8	3
Man5-Man9	1	1	0	0	0	0	10
other N-glycans	2	6	4	8	1	1	10
total	100	100	100	100	100	100	100

**FIGURE 3:**
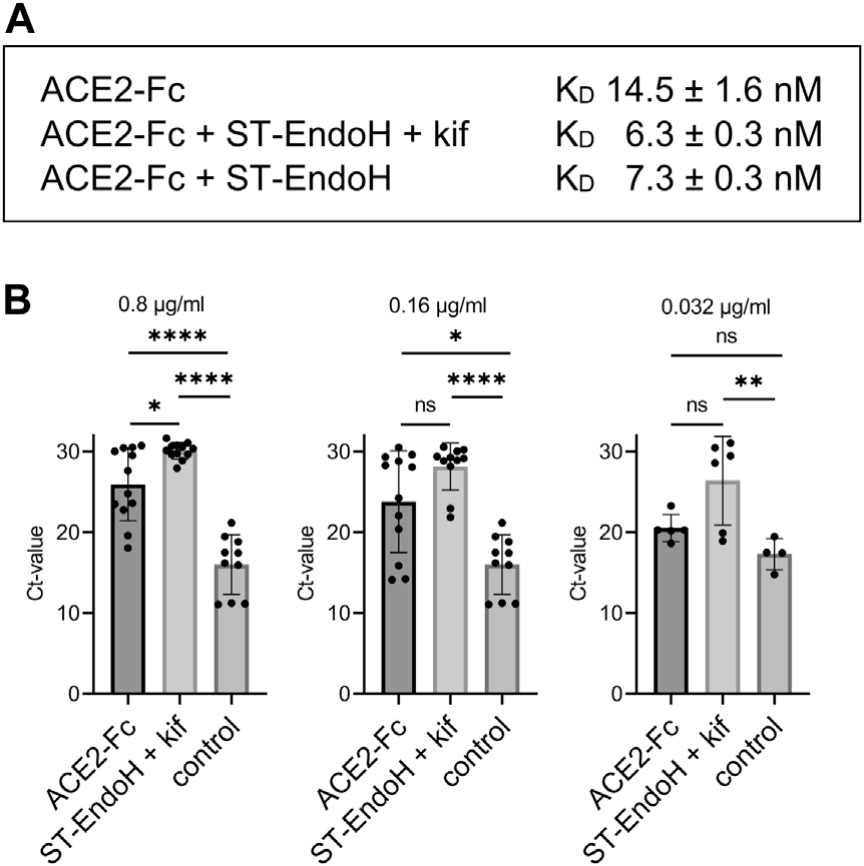
RBD binding and SARS-CoV-2 neutralization by ACE2-Fc. **(A)** Binding of RBD-His to immobilized *N. benthamiana* ACE2-Fc determined by SPR. Values are mean ± SD (n = 4). **(B)** Virus neutralization assay: SARS-CoV-2 (Delta variant) was mixed with the indicated concentrations of recombinant ACE2-Fc and added to Calu-3 cells. Viral RNA was extracted from cell culture supernatant after 24 h and quantified by RT-qPCR. Control: infected cells without neutralizing agent. Data are presented as mean ± SD (n = 4 to 12; “ns” not significant, “*” *p*-value <0.05, “**” *p*-value <0.01, “****” *p*-value <0.0001 according to one-way ANOVA).

To see if the produced proteins are functional, we analyzed the binding of ACE2-Fc to RBD by SPR. Consistent with findings for mammalian cell-produced ACE2 (Capraz et al., 2021), the K_D_ value was lower for the *in vivo* trimmed ACE2-Fc variants produced with or without addition of kifunensine (Figure 3A and Supplementary Figure S1). Next, we compared ACE2-Fc with GnGn N-glycans and ACE2-Fc with “single GlcNAc” N-glycans in a SARS-CoV-2 live virus neutralization assay using the Delta variant. At all tested ACE2-Fc concentrations, plant-produced ACE2-Fc with N-glycans consisting of single GlcNAc residues displayed higher virus neutralization activity compared to plant-produced ACE2-Fc with GnGn structures (Figure 3B). Together, these data show that the *in vivo* “deglycosylation” approach described in this study allows the production of a potentially superior ACE2-Fc decoy receptor in plants.

## 4 Discussion

Engineered decoys that closely mimic viral cell entry receptors are very promising candidate therapeutics to combat diseases that are caused by continuously mutating viruses. Various ACE2 decoys based on different designs have been described since the beginning of the SARS-CoV-2 pandemic (Chan et al., 2020; Arimori et al., 2022) and functional human ACE2 decoys were successfully produced in plants (Siriwattananon et al., 2020; Castilho et al., 2021; Daniell et al., 2022). An *in planta* deglycosylation approach was already previously applied to produce glycoengineered ACE2 (Mamedov et al., 2021). In this study, a poly-histidine-tagged ACE2 variant was retained in the ER together with EndoH to facilitate the cleavage of mannosidic N-glycans from ACE2. While binding of the glycosylated and deglycosylated ACE2 variants to RBD was comparable, the EndoH-coexpressed ACE2 was far less potent than its glycosylated counterpart in a virus neutralization assay. These data are contrary to our findings and the data from *in vitro* deglycosylation of mammalian cell-produced ACE2 variants (Capraz et al., 2021). The reason for the discrepancy is currently unknown, but we speculate that the premature removal of mannosidic N-glycans in the ER could have some adverse effect on ACE2 folding. In our approach, ST-EndoH-GFP likely acts after the majority of the protein is properly folded and released from lectin-based quality control in the ER. Our data suggest that ST-EndoH-GFP is already partially active in the *cis*/medial-Golgi where Golgi α-mannosidase I trims mannose residues to enable the initiation of complex N-glycan formation by N-acetylglucosaminyltransferase I (GnTI) (Strasser et al., 2021). Overexpressed ST-EndoH-GFP on its way to the *trans*-Golgi or upon recycling from the *trans*-Golgi to the *cis*/medial-Golgi could compete with Golgi α-mannosidase I for substrates, which would explain the considerable amounts of ACE2 “single GlcNAc” N-glycans generated in the absence of the α-mannosidase inhibitor kifunensine.

The increased virus-neutralization activity of plant-produced ACE2-Fc carrying GnGn N-glycans as compared to its counterpart derived from human cells (Castilho et al., 2021) is likely attributed to the absence of terminal sialic acid on N-glycans attached to N90 and N322, which interfere with spike binding (Capraz et al., 2021). The removal of most ACE2 N-glycans by EndoH *in planta* further increased the affinity to RBD. Moreover, we showed here that the ACE2-Fc with “single GlcNAc” N-glycans displays increased virus neutralization. While enzymatic deglycosylation of purified ACE2 decoys *in vitro* is in principle feasible (Capraz et al., 2021), the *in planta* N-glycan removal procedure appears more straightforward and could lead to a more affordable product.

Deglycosylated soluble ACE2 decoys with enhanced properties have also been generated in mammalian cells by mutating N-glycosylation sites like N90 or N322 or combinations thereof (Capraz et al., 2021; Isobe et al., 2022). Of note, mutation of the N53 site reduced the affinity to RBD and virus uptake and attenuated the enhancing effect of the N90 and N322 mutations suggesting that site-specific removal of N-glycans could result in an optimized ACE2-decoy. However, these approaches may interfere with protein folding and involve the introduction of non-natural mutations which raises safety concerns when recombinant ACE2 decoys are administered in high concentrations to humans (Arimori et al., 2022). Whether the “single GlcNAc” N-glycans on ACE2-Fc are preferable in this aspect or also lead to unwanted immunological side effects remains to be investigated. In summary, our data show that a potentially superior soluble ACE2-Fc decoy with homogeneous N-glycans consisting of single GlcNAc residues can be generated *in planta* by co-expression of a Golgi-targeted endoglycosidase.

## Data Availability

The mass spectrometry proteomics data have been deposited to the ProteomeXchange Consortium via the PRIDE (Perez-Riverol et al., 2022) partner repository with the dataset identifier PXD041320. Other data that support the findings of this study are available from the corresponding author upon request.
