# Supplementary material for: In planta deglycosylation improves the SARS-CoV-2 neutralization activity of recombinant ACE2-Fc
Source: Front Bioeng Biotechnol. 2023 May 3;11:1180044. doi: 10.3389/fbioe.2023.1180044 (PMC10190127; doi:10.3389/fbioe.2023.1180044)
Supplement: Supplementary file 1 [file DataSheet1.PDF]

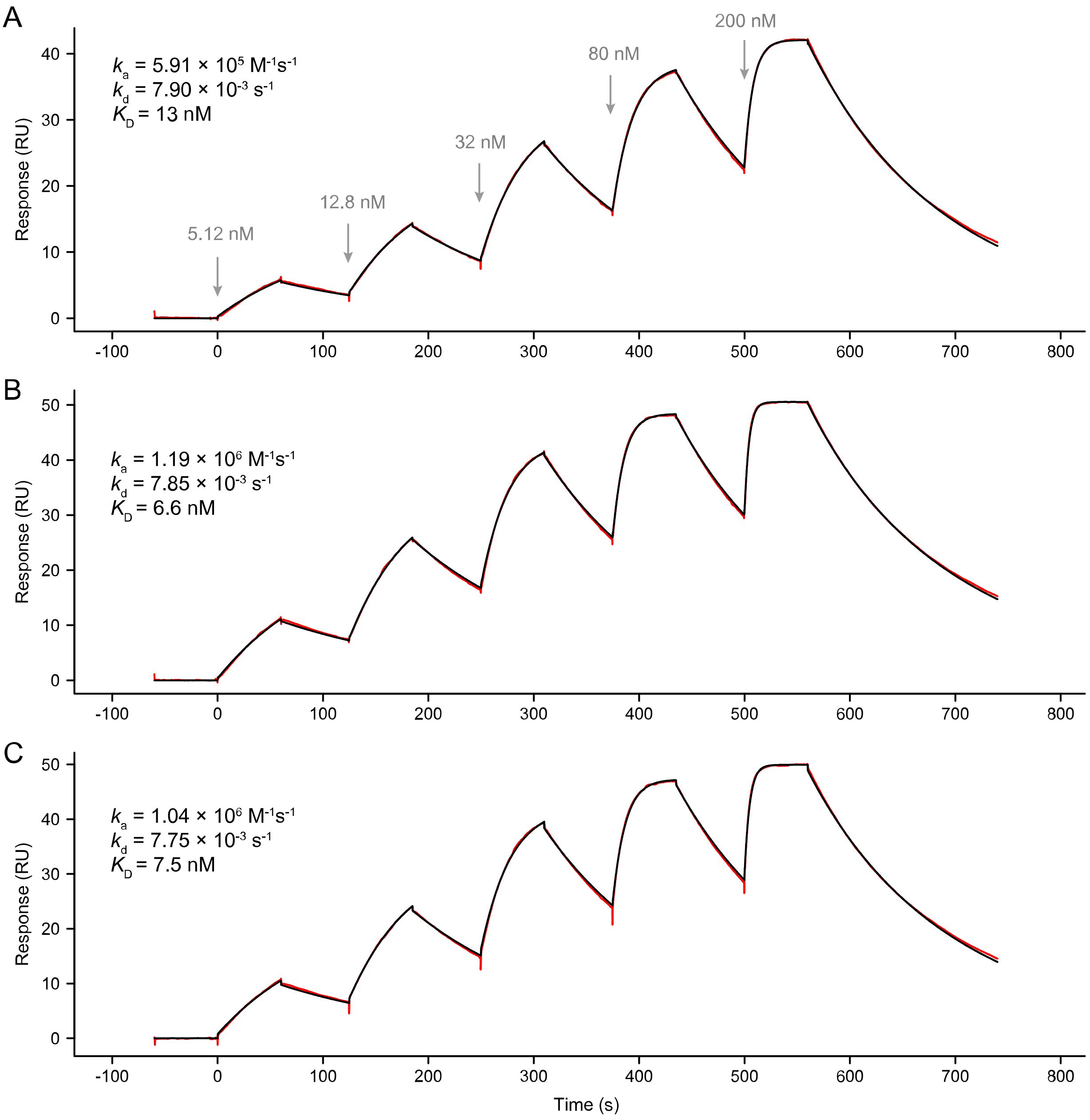

Supplementary Figure 1. Representative SPR sensorgrams of RBD-His interacting with ACE2-Fc variants produced in *N. benthamiana* and immobilized onto the sensor chip surface. Double reference subtracted curves of the kinetic titration series are shown in red, and the 1:1 model fit is represented in black. ACE2-Fc was either expressed alone (**A**) or in the presence of ST-EndoH with (**B**) or without (**C**) kifunensine.
